# Bevacizumab and Weekly Docetaxel in Patients with Metastatic Castrate-Resistant Prostate Cancer Previously Exposed to Docetaxel

**DOI:** 10.1155/2011/258689

**Published:** 2011-08-21

**Authors:** Filippo Francini, Alessandra Pascucci, Edoardo Francini, Gianluca Bargagli, Raffaele Conca, Antonella Licchetta, Giandomenico Roviello, Ignazio Martellucci, Giorgio Chiriacò, Salvatora Tindara Miano, Giuseppe Marzocca, Antonio Manganelli, Roberto Ponchietti, Vinno Savelli, Roberto Petrioli

**Affiliations:** ^1^Department of Odontostomatology and Maxillo-Facial Surgery, University of Siena, Policlinico Le Scotte, Viale Bracci 11, 53100 Siena, Italy; ^2^Medical Oncology, University of Siena, Policlinico Le Scotte, Viale Bracci 11, 53100 Siena, Italy; ^3^General Surgery, University of Siena, Policlinico Le Scotte, Viale Bracci 11, 53100 Siena, Italy; ^4^Urologic Surgery, University of Siena, Policlinico Le Scotte, Viale Bracci 11, 53100 Siena, Italy; ^5^Genitourinary Unit, University of Siena, Policlinico Le Scotte, Viale Bracci 11, 53100 Siena, Italy; ^6^Department of Surgery, University of Siena, Policlinico Le Scotte, Viale Bracci 11, 53100 Siena, Italy

## Abstract

*Background*. The aim of this paper was to evaluate the activity and tolerability of docetaxel (D) and bevacizumab (Bev) in patients with metastatic castrate-resistant prostate cancer (CRPC) previously exposed to D. *Methods*. Treatment consisted of D 30 mg/m^2^ i.v. for four consecutive weekly administrations followed by a 2-week rest interval, in addition to Bev 5 mg/kg i.v. every 2 weeks. *Results*. Forty-three patients were enrolled: a PSA response was observed in 27 patients (62.7%, 95% CI: 0.41 to 0.91), and a palliative response was achieved in 31 patients (72.1%, 95%CI: 0.48 to 1.02). After a median followup of 11.3 months, only five patients had died. The regimen was generally well tolerated. *Conclusion*. Weekly D + biweekly Bev seems to be an effective and well-tolerated treatment option for patients with metastatic CRPC previously exposed to D-based chemotherapy.

## 1. Introduction

The results of two large randomised trials have provided substantial support in favor of the role of chemotherapy in the treatment of castrate-resistant prostate cancer (CRPC) by demonstrating that docetaxel (D) and prednisone (P) improve survival in comparison with older regimens and significantly improve the quality of life [1, 2]. Therefore, D has become the first-line standard of care for metastatic CRPC, with PSA responses of about 50% and median survivals of usually less than 20 months. 

Patients with CRPC who progress after D treatment may be considered for a second-line chemotherapy, especially if they have a reasonable performance status, have symptoms, and/or are likely to soon develop symptoms for their disease. In this setting, a recent randomized phase III trial demonstrated that cabazitaxel, a tubuline-binding taxane drug, improved survival in metastatic CRPC patients with progressive disease after D-treatment, with a 30% reduction in the risk of death compared with mitoxantrone taken as control group [3].

D resistance is a common problem in the treatment of many tumors including CRPC, and the development of new drugs that may overcome such resistance is important to extend D activity [4]. 

Angiogenesis is an important process for growth, progression, and metastasis of solid tumors, and the inhibitor of the vascular endothelial growth factor (VEGF) bevacizumab (Bev) is currently approved for the treatment of colon, lung, breast, and clear cell renal carcinoma in the metastatic setting [5]. 

In addition, preclinical data demonstrated that VEGF inhibition may also prevent further tumor growth of the prostate cancer cell line DU 145 implanted in nude mice, and preliminary clinical studies suggested that Bev combined with chemotherapy is tolerable and has promising activity in CRPC patients [6, 7]. 

Although Bev achieved no PSA response when it was used in monotherapy, interesting results were reported by the combination of Bev with D and estramustine as first-line treatment in a previous study of the Cancer and Leukemia Group B [8]. Moreover, a recent study described promising data in terms of PSA response and objective response in pretreated patients with CRPC receiving D and Bev [9]. On these previous experiences, and in the hypothesis that Bev may overcome the resistance to D, we tested the activity and tolerability of Bev combined with D in CRPC patients with disease progression during or after D-based first-line chemotherapy.

## 2. Patients and Methods

### 2.1. Eligibility Criteria

This phase II study involved patients with histologically confirmed, measurable, or evaluable advanced prostatic adenocarcinoma who had progressed while on D or within 60 days after the last D dose. This last eligibility criterium, together with a minimum of 3 months of D-based chemotherapy as first-line treatment, was required in order to better elucidate the benefit of the addition of Bev. Patients were admitted to the chemotherapy protocol provided that they met at least one of the following criteria: a positive bone scan a ≥25% increase in PSA (PSA higher than 2 ng/mL) in comparison with baseline on two successive occasions separated by at least two weeks for patients without measurable disease; new metastatic lesions revealed by a bone scan; and a ≥25% increase in a bidimensionally measurable tumor mass. All of the patients had to have an Eastern Cooperative Oncology Group (ECOG) performance status (PS) of ≤ 2, adequate hematological (leukocytes ≥ 3000/mm^3^; hemoglobin ≥ 10 g/dL, platelets ≥ 100,000/mm^3^), renal (serum creatinine ≤ 2.0 mg/dL), and hepatic function (serum bilirubin ≤2.0 mg/dL; Table 1).

Patients were excluded if they had not received prior D-based chemotherapy or if they had congestive heart failure, a recent myocardial infarction, or any other previous malignant diseases except basal cell carcinoma of the skin. Bisphosphonates were admitted in all of patients who presented with bone metastases.

The study was approved by the ethical committee of Siena University, and all patients provided their written informed consent. 

### 2.2. Treatment Plan

Treatment consisted of D 30 mg/m^2^ as a 30-minute intravenous infusion, using a schedule of four consecutive weekly administrations followed by a 2-week rest interval, in addition to Bev 5 mg/kg intravenously every 2 weeks. Premedication consisted of P 10 mg p.o. (12 h before, at the time of, and 12 h after D administration). Cycles were administered if serum leukocytes were ≥3000/mm^3^, granulocytes > 1500/mm^3^, and platelets > 100,000/mm^3^. Ondansetron 8 mg was administered at the beginning of each treatment cycle as antiemetic medication. The patients continued to take analgesic medication at doses adjusted to provide optimal pain control. The chemotherapy was administered until disease progression or unacceptable toxicity, and for a maximum of 30 weekly D cycles. In responding patients, Bev could be continued at the investigator's discretion, or until disease progression or unacceptable toxicity. 

### 2.3. Response Assessments

Tumor response in patients with measurable lesions was evaluated using the RECIST criteria [10]. Serum PSA was measured every three weeks: a PSA response was defined as a reduction from baseline of at least 50% for at least three weeks whereas PSA progression was defined as an increase from nadir of at least 25% and ≥2 ng/mL [11]. Pain symptomatology was measured at baseline and then every 6 weeks by the McGill Melzack Pain Questionnaire, and pain response was defined as a 2-point reduction in the 6-point present pain intensity scale (or the complete disappearance of pain if the initial score was 1+) [12]. These results had to be maintained at two consecutive evaluations made at least 3 weeks apart and without any increase in analgesic consumption. The patients were asked to classify the average pain level during the previous 24 h. We used a translated form of the McGill Melzack Questionnaire to which the “reconstruction-based methodology” has been applied [13]. Analgesic consumption was based on the average daily quantities taken by the patient during the previous week, and assigned oral morphine equivalents before analysis [14]. 

The laboratory studies (blood and platelet counts, and a comprehensive screening profile) were performed at baseline and every three weeks, and the patients underwent a weekly complete blood cell count and electrolytes profile before chemotherapy. 

The imaging studies included abdominal and pelvic CT or magnetic resonance imaging, a bone scan, and chest radiography. All measurable diseases were reevaluated at 8-week intervals. Radionuclide bone scans were repeated after 3 months. In all subjects, fasting venous blood samples were drawn between 8.00 and 9.00 a.m. after a 12-h fasting period at baseline and after 3 months in order to assess the bone resorption marker crosslinked C-terminal telopeptide (CTX) and the bone formation marker bone alkaline phosphatase (B-ALP). 

In all cases, a baseline ECG was obtained, and a further cardiac work-up was performed if indicated. Bone disease progression was defined as the appearance of any new bone lesion or the progression of existing bone metastases. A dental examination, including orthopantomography (OPT), was performed in all patients at baseline, and active dental surveillance every three months.

### 2.4. Treatment-Related Adverse Events

Toxicity was defined using the National Cancer Institute (NCI) Common Toxicity Criteria, version 3.0. The treatment was delayed at the first occurrence of grade II hematological toxicity, and administered at the same dose after it returned to grade I or better. In the case of grade III or IV toxicity, the treatment was interrupted and a maximum of three weeks was allowed for recovery, after which the patients were withdrawn from the study. In the case of a second episode of grade III or IV toxicity in the same patient, treatment was resumed after recovery and the subsequent administration of D was reduced to 20 mg/m^2^. Chemotherapy protocol was discontinued if the ejection fraction decreased below the institutional lower limit of normal and declined by ≥15%.

### 2.5. Statistical Considerations

The primary endpoint was PSA response. In accordance with Simon's “optimal design”, a sample size of 36 patients was planned, assuming a response rate of approximately 10% for other second-line chemotherapies, and a target level of interest of 30%, with an *α* of 0.05 and a *β* of 0.90. In the hypothesis of 10%–20% inevaluable patients, about 40 patients were planned to be enrolled to better estimate the response. Secondary endpoints were pain response, progression-free survival (PFS), and overall survival. PFS was defined as the time from starting chemotherapy to the first occurrence of objective or PSA progression, or death due to any cause.

## 3. Results

From September 2008 to April 2010, 43 patients were enrolled. Their median age was 74 years (range 58–82 years) Thirty-seven patients had bone metastases. and seventeen patients had measurable disease (Table 1). Most of enrolled patients have participated in a randomized phase II study which compared the combination of weekly D and weekly Epirubicin (EPI) with the conventional 3-weekly D [15]. All patients who had achieved a response or a stable disease during first-line chemotherapy had been retreated with D-based chemotherapy. The median dose of D received before the enrollment in the current study was 940.8 mg/m^2^ (range 30–1122,3).

All enrolled patients were treated with the new treatment regimen within 60 days from the end of last D dose (range 12 to 52 days). Two patients received only one weekly chemotherapy cycle for treatment-unrelated reasons. Two patients were lost to followup after four and six months from the start of treatment. All patients were included in the overall analysis (intent-to-treat). A total of 968 weekly D cycles (median 21, range 11–30) and a total of 1172 biweekly cycles of Bev (median 26, range 6–41) were administered. 

### 3.1. Biochemical Response

**Figure 1:**
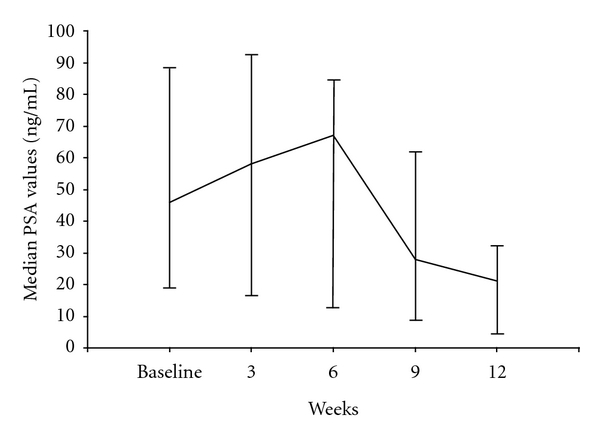
Median PSA (with minimum and maximum values) in 18 out of 27 responding patients who showed a PSA surge within the first 3 months of treatment with weekly D + biweekly Bev.

A decrease in PSA levels >50% was observed in 27 patients (62.7%, 95% CI: 0.41 to 0.91), and nine patients (20.9%) had stable PSA for at least twelve weeks (Table 3). After the first 3 weekly cycles a PSA surge was observed in 18 out of 27 responding patients: in all these patients PSA then progressively decreased and at the third month was less than 50% with respect to the baseline values (Figure 1). 

During the prior first-line chemotherapy, 15 out of the 27 responding patients had achieved PSA response while 8 had achieved stable disease and 4 patients had progressed.

### 3.2. Objective Response

Of seventeen patients with measurable disease, eight achieved PR and seven had stable disease: objective responses were observed on prostate cancer (3 cases), prostate cancer and pelvic lymph nodes (3 cases), and prostate cancer and lung metastases (2 cases). 

The bone scan, which could be repeated after 3 months of treatment in 35 out of 37 patients with bone metastases, showed stable disease in 29 patients, and partial remission in 5 patients; two or more new lesions compared with the prior scan for trial entry were described in one patient. This same patient had PSA progression after 3 months and chemotherapy was stopped. The bone markers CTX and B-ALP were reduced >50% with respect to baseline values in 33 and 28 patients, respectively, after 12 weeks from the start of treatment (65% median reduction for CTX and 58% median reduction for B-ALP). A palliative response was observed in 31 patients after 12 weeks (72.1%, 95% CI: to 0.48 to 1.02). 

After 12 weeks from the start of the new treatment protocol, seven patients (including two subjects who received only one treatment cycle) had PSA progression, but three of them had a reduction in bone pain with decrease in analgesics use, improvement in performance status, and reduction in serum levels of the bone markers CTX and BALP. Despite the initial end-point of the study, because of the achieved clinical benefit, our oncology group and the scientific ethical committee decided to continue D + Bev in these three patients until worsening of pain and/or performance status.

After a median followup of 11.3 months, 18 patients showed PSA progression and only five patients had died.

The regimen was generally well tolerated, and no unexpected toxic effects were observed (Table 4). No grade 4 toxicity or congestive heart failure was observed, and all cycles were administered on an outpatient basis. The most frequent side effects were neutropenia, anemia, thrombocytopenia, epistaxis, and fatigue, which were grade I or II in most cases. Grade III fatigue was observed in two patients after nine and sixteen cycles, respectively: despite the reduction of D dose and the discontinuation of Bev, treatment was then interrupted in these cases because of the persistence of this side effect. Grade 1 epistaxis was observed in 23 patients (53.4%) during treatment, but reached grade 2 in only 7 cases and grade 3 in one patient. No patient developed osteonecrosis of the jaw (ONJ). Dose reduction of D was required in a total of twelve patients: 37 (3.8%) weekly D cycles were administered with a 33% reduction, down to 20 mg weekly. A total of 57 (5.8%) weekly D cycles were delayed: the reason for the delays were hematological in 41 (71.9%) and nonhematological in 16 (28.1%) cycles. A total of 46 (39.2%) biweekly Bev cycles were delayed: the reasons for the delays were haematological in 39 (84.8%) and non-hematological in 7 (15.2%) cycles.

## 4. Discussion

The currents phase II study is the first extended report which suggests that the combination of weekly D with the biweekly administration of the antiangiogenic agent Bev is effective and tolerable in the treatment of patients with metastatic CRPC who have progressed after D-based chemotherapy: the 62.7% PSA and 72.1% palliative response compare favorably with the results observed in phase II studies of second-line chemotherapy [16, 17]. Other chemotherapeutic agents might be used after initial treatment with D, including old drugs such as vinorelbine, oral cyclophosphamide, etoposide, mitoxantrone, vinblastine, and doxorubicin, but most studies reported no objective response and less than 15% laboratory response [18]. Other studies described modest activity with satraplatin, a third-generation platinum analog, or ixabepilone, an epothilone, with mitoxantrone, or also with the combination of D and high-dose calcitriol [19–21]. 

It must be remembered that in most clinical trials a few patients stop the first-line treatment with D while still responding to the drug. In this group of patients a repeated treatment with D might be appropriated if progression occurs after a reasonably long time interval [22]. In our study, the strict eligibility requirement of progression while on D or within 60 days of the last D dose means that these patients might have not responded to rechallenge with D. Notably, responses were seen also in patients who had not shown an initial response to prior D as first-line treatment. Therefore, this finding and the characteristics of enrolled patients support an effective role of Bev in restoring the sensitivity to D and also in reversing resistance in patients who were previously nonresponders to the drug (Table 1). Bev is able to alter tumor vasculature, for example, decreasing tumor vessel permeability and increasing intratumoral perfusion, which might turn into an improved tumor delivery of a cytotoxic agent, thus enhancing its antitumor activity [23]. Since the limited tissue penetration is an important mechanism of tumor resistance to taxanes, the effects of the antiangiogenic agent Bev may be a possible explanation of the observed reversal of D resistance in our population study [24].

Another point to consider is the observed PSA surges during treatment protocol in 18 out of 27 responding patients, which may suggest massive cancer cell death and PSA release, thus indicating efficacy, as also reported in other previous trials during chemotherapy for CRPC [25, 26]. Nevertheless, despite the unknown biological relevance of this transient initial PSA increase, most of our patients achieved an improvement in bone symptomatology and in performance status, and PSA decreased > 50% at 3 months (Figure 1). Furthermore, PSA results correlated with changes in bone markers, since CTX and B-ALP were reduced with respect to baseline values in all these patients, and this was probably related to the real antitumor activity of the D + Bev combination.

Notably, despite PSA progression after 3 months of treatment, three patients continued to have an improvement in performance status and reduction in bone symptomatology and bone markers, and because of this achieved clinical benefit they continued to receive D + Bev. This finding may confirm that the progression criteria that are usually suitable for assessment of efficacy of cytotoxic agents in CRPC may not be suitable for discriminating treatment effects of targeted agents such as Bev [27, 28]. It may be that significant treatment benefits with targeted therapies need long time scales to emerge, possibly due to its noncytotoxic-targeted mechanism of action. 

As Bev-based salvage treatment, a 55% PSA response and 37.5% objective response was found in 20 pretreated patients with CRPC receiving D 60 mg/m^2^ and Bev 10 mg/kg every 3 weeks [9]. Another recent experience suggested a benefit in progression-free and in overall survival by the use of weekly D 25 mg/m^2^ combined with Bev 10 mg/kg every two weeks in CRPC patients [29]. The toxicity profile of our treatment protocol was comparable to that observed in these BEV-based salvage treatments, with a major incidence of grade IV neutropenia and thrombocytopenia reported by the use of 3-weekly D schedule. Considering the strict eligibility criteria of our study and the fact that all our patients had previously received at least two chemotherapy lines, the current results appear even more encouraging than that found in the aforementioned reports. Nevertheless, it must be considered that most of metastatic CRPC patients who relapse after the first line D and D rechallenge do not survive more than 6 months. It is notable that in the current study, after a median follow-up time of 11.3 months, only five patients had died and most patients who started the new treatment protocol more that 12 months ago are still alive and have a good quality of life. 

Therefore, although three-weekly D and P remains the conventional treatment protocol in first-line setting, it may be hypothesized that weekly D combined with biweekly Bev, as applied by us, is an appropriate schedule in terms of activity and toxicity for heavily pretreated patients. The combination of weekly scheduling of D with Bev has shown interesting activity without significant toxicity also in breast, ovarian and mesenchymal tumors [30–32].

The efficacy and safety results of the current study compare favorably also with those reported with the drug cabazitaxel, that was recently approved by US Food and Drug administration for second-line treatment of metastatic CRPC patients [3]. Severe neutropenia was common in cabazitaxel trial (89%), and 18% of patients discontinued the study treatment because of adverse events while grade III neutropenia was observed in only 18.6% of cases in our population study. Nevertheless toxicity was mild in our patients: adverse events likely related to Bev (hypertension, epistaxis, and albuminuria) never reached grade 3 and were easily manageable, as usually reported in other tumors with the biweekly schedule of 5 mg/kg of Bev. The low toxicity of D was mainly due to the weekly schedule. The efficacy of weekly D seems to be similar to that of the usual 3-weekly schedule, but their comparative toxicities differ markedly, with moderate to severe myelosuppression being common when the drug is administered once every 3 weeks [33]. As well as is concerned other adverse events, Altough the new and potent antiangiogenic therapies might theoretically enhance the antiangiogenic effects of zoledronic acid on bone tissue, our findings do not suggest a trend for a possible higher incidence of bisphosphonate-induced ONJ for patients receiving zoledronic acid and Bev [34].

Another point to consider is that the percentage of enrolled patients ≥75 years was about 50% in the current study, compared with only 18% in cabazitaxel trial. On these findings, it seems that weekly D and biweekly Bev can be safety administered also to elderly patients, who represent the most part of CRPC population. Therefore, although cabazitaxel will be the only established second-line treatment of CRPC patients in the next future, weekly D and Bev may be a valid option for patients with a decreased hematological reserve and/or for elderly subjects. Moreover, in the absence of a randomized comparison between cabazitaxel and our proposed treatment protocol, weekly D and Bev might be used after cabazitaxel failure.

In conclusion the results of this study suggest that weekly D and biweekly Bev is an effective and well-tolerated treatment option for patients with metastatic CRPC previously exposed to D. Bev seems to overcome the resistance to the drug in patients who had progressed during or after D-based chemotherapy.

## 5. Conclusion

Weekly D + biweekly Bev seems to be an effective and well-tolerated treatment option for patients with metastatic CRPC previously exposed to D-based chemotherapy.

## Figures and Tables

**Table 1:** Main eligibility criteria.

Histologically confirmed advanced prostatic carcinoma	
Progression while on D or within 60 days after the last D dose	
A positive bone scan and a ≥25% increase in PSA in comparison with baseline	
New metastatic lesions revealed by a bone scan	
A ≥25% increase in bidimensionally measurable tumor mass	
ECOG PS of ≤2	
Leukocytes ≥3000/mm^3^, haemoglobin ≥ 10 g/dL, platelets ≥ 100.000/mm^3^	
Serum creatinine ≤ 2.0 mg/dL; serum bilirubin ≤ 2.0 mg/dL	

**Table 2:** Main patient characteristics.

Enrolled patients	43
Median age (range): years	74 (58–82)
≥75 years	21 (48.8%)

ECOG performance status	
0	9
1-2	34

Sites of metastases	
Bone	26
Bone + prostate cancer	4
Bone + prostate cancer + lymph nodes	4
Bone + lung	3
Prostate cancer + lymph nodes	3
Liver + lymph nodes	2
Prostate cancer + lung	1

Median PSA (range), ng/mL	78 (47–374)

Previous treatment	
Prostatectomy	31
Radiotherapy	9

Hormone therapy	
1	28
≥2	15

Prior first-line chemotherapy	
w-Epirubicin + w-docetaxel	21
3-w Docetaxel + prednisone	15
w-Docetaxel + prednisone	7

Number of chemotherapy regimens	
2	43
>2	23

Prior third-line chemotherapy	
Docetaxel + prednisone	23

Best response to prior first-line chemotherapy	
PSA decline ≥50%	31
Stable disease	7
Progressive disease	5
Baseline pain intensity	
0	0
1	8
2	22
3	8
4	5
5	0

Median hemoglobin, g/dL	10.4
Range	7.9–13.8

**Table 3:** Response to treatment.

Enrolled patients	43
Biochemical response	
PSA decline ≥50%	27 (62.8%)
Stable disease	9 (20.9%)
Progressive disease	7 (16.3%)

Objective response	
Partial remission	8/17 (47.1%)
Stable disease	7/17 (41.1%)
Progressive disease	2/17 (11.8%)

Palliative response	31 (72.1%)

**Table 4:** Number of patients experiencing the most frequent treatment-related adverse events.

	Grade 1	Grade 2	Grade 3
Hematological			
Neutropenia	19 (44.1%)	14 (32.5%)	8 (18.6%)
Anemia	20 (46.5%)	15 (34.8%)	6 (13.9%)
Thrombocytopenia	18 (41.8%)	12 (27.9%)	4 (9.3%)

Nonhematological			
Nausea/vomiting	12 (27.9%)	8 (18.6%)	0
Diarrhea	9 (20.9%)	6 (13.9%)	0
Constipation	13 (30.2)	11(25.5%)	0
Nail changes	22 (51.1% )	17 (39.5%)	2 (4.6%)
Dry eye/tearing	26 (60.4%)	15 (34.8%)	0
Myalgia/arthralgia	22 (51.1%)	14 (32.5%)	0
Fatigue	21 (48.8%)	18 (41.8%)	2 (4.6%)
Sensory neuropathy	16 (37.2%)	7 (16.2%)	0
Peripheral edema	22 (51.1%)	8 (18.6%)	0
Epistaxis	23 (53.4%)	7 (16.2%)	1 (2.3%)
Dyspnea	12 (27.9%)	5 (11.6%)	0
